# Dual Synthetic Pathways for Organotin-Functionalized Mesoporous Silica Nanoparticles: Targeted Therapeutic Platforms with Folic Acid and PEI Formulation

**DOI:** 10.3390/nano15231791

**Published:** 2025-11-27

**Authors:** Victoria García-Almodóvar, Sanjiv Prashar, Santiago Gómez-Ruiz

**Affiliations:** 1 COMET-NANO Group, Departamento de Biología y Geología, Física y Química Inorgánica, E.S.C.E.T., Universidad Rey Juan Carlos, Calle Tulipán s/n, Móstoles, E-28933 Madrid, Spain; victoria.galmodovar@urjc.es (V.G.-A.); sanjiv.prashar@urjc.es (S.P.); 2 Instituto de Investigación de Tecnologías para la Sostenibilidad, Universidad Rey Juan Carlos, Calle Tulipán s/n, Móstoles, E-28933 Madrid, Spain

**Keywords:** breast cancer, nanoparticles, polyethyleneimine, folic acid, tin

## Abstract

Breast cancer is the most common cancer in women worldwide, with a high mortality rate. Moreover, the treatments currently used to address this disease are sometimes ineffective and cause numerous side effects. For this reason, the search for new treatments that can overcome these challenges is a growing field of research. One potential solution under investigation is the use of mesoporous silica nanoparticles (MSNs). These materials possess excellent properties, making them attractive as starting platforms for various compounds. In this study, different compounds with distinct properties were anchored onto these nanoplatforms. The first is polyethyleneimine (PEI), which, when formulated within the nanoparticle, increases its bioavailability. The second is folic acid (FA), a molecule that enables active targeting of tumor cells. Finally, an organotin(IV) complex was incorporated via two different anchoring strategies to provide therapeutic action. This multifunctional platform thus combines three activities simultaneously. MTT assay studies revealed that the final material, MSN-TEDTH-PEI-FA-TR-Sn, demonstrates potential against the MCF-7 tumor cell line while showing no toxicity to the healthy Hek 293T cell line. These findings make it an interesting candidate for future *in vivo* trials.

## 1. Introduction

The development of advanced nanomaterials for biomedical applications has revolutionized the landscape of disease diagnosis and treatment, offering solutions that mitigate the adverse effects of conventional therapies [1,2]. Among the wide array of nanomaterials with biomedical applications, inorganic nanomaterials stand out as a versatile and promising category [3,4,5,6]. Within this group, a diverse range of materials can be classified based on their composition, morphology, or pore distribution [7,8]. One of the most extensively studied and utilized materials is mesoporous silica nanoparticles (MSNs), thanks to their remarkable physicochemical properties and the ease with which they can be manipulated and functionalized. MSNs exhibit several outstanding characteristics, including high chemical and thermal stability, a large specific surface area, tunable pore size, uniform pore distribution, and excellent biocompatibility [9,10]. These features make MSNs exceptional candidates as carriers for therapeutic agents and other molecules of interest [11,12]. Moreover, they enable the integration of multiple functionalities into a single nanoparticle, offering unprecedented versatility. Given these advantages, MSNs are particularly attractive for applications in cancer treatment, where they aim to address the limitations of conventional therapies such as chemotherapy [13,14]. Their ability to deliver drugs in a targeted and controlled manner while minimizing side effects has positioned them as a cornerstone in the development of next-generation therapeutic platforms [15,16,17].

Breast cancer is one of the most prevalent and deadly cancers affecting women worldwide, responsible for over 666,000 deaths in 2022 alone [18]. This disease poses significant therapeutic challenges due to its heterogeneity and its tendency to develop resistance to conventional treatments such as chemotherapy, making it imperative to explore alternative strategies [19,20]. In this context, mesoporous silica nanoparticles (MSNs) functionalized with specific targeting and therapeutic agents emerge as a promising platform for precision oncology and the development of personalized treatments [21,22]. A commonly used cell line model in breast cancer research is MCF-7, derived from the pleural effusion of a 69-year-old Caucasian woman diagnosed with metastatic breast adenocarcinoma. These cells are frequently employed to study cytotoxicity and the therapeutic effects of experimental treatments [23]. On the other hand, to evaluate systemic toxicity, the human embryonic kidney cell line Hek 293T is often used. This is particularly relevant as the kidneys serve as a major route for the elimination of therapeutic agents [24]. This dual-cell line approach enables a comprehensive evaluation of the impact of these nanoparticles, allowing for the assessment of their effects on both malignant and healthy tissues. By integrating these models, researchers can better understand the therapeutic potential and safety profile of MSNs in breast cancer treatment [25].

One of the major drawbacks of conventional treatments is their lack of specificity, which leads to a high incidence of side effects in healthy tissues and organs [26]. To address this issue in nanoparticle-based therapies, a promising strategy involves their functionalization with folic acid (FA). This molecule is particularly appealing due to its ability to enhance treatment selectivity [27]. This selectivity arises from the overexpression of folate receptors in cancer cells; a characteristic commonly observed in breast cancer and other malignant neoplasms [28].

By leveraging this overexpression, FA-functionalized MSNs can selectively accumulate in tumor tissues, significantly enhancing therapeutic efficacy while minimizing systemic side effects [29,30]. This targeted approach not only improves the precision of the treatment but also reduces the collateral damage associated with traditional cancer therapies, offering a compelling alternative for more effective and safer treatments [31].

It is essential for the nanoplatform to include a compound capable of performing the therapeutic function [32]. For this reason, an organotin(IV) compound (Sn) is incorporated into the MSNs. Organotin compounds are well known for their potent antitumor properties, including their ability to induce apoptosis and disrupt cellular signaling in malignant cells [33]. However, their clinical application has been hindered by challenges such as poor solubility, rapid degradation, and non-specific toxicity [34]. By incorporating these compounds into MSNs, their stability, solubility, and bioavailability can be significantly improved, effectively mitigating these limitations and enabling more controlled therapeutic outcomes [35,36,37]. Moreover, organotin compounds are known to circumvent tumor cell resistance a major drawback of platinum-based complexes commonly used in chemotherapy [38,39]. This characteristic positions organotin-functionalized MSNs as a promising alternative for overcoming drug resistance and enhancing the effectiveness of cancer treatments.

Until now, we have highlighted the excellent properties of MSNs. However, it is important to acknowledge a minor drawback: their occasionally low dispersibility in challenging biological environments [40,41]. To address this limitation, the external surface of MSNs is modified with a polyethyleneimine (PEI) formulation. This cationic polymer enhances the functionality of MSNs by providing several key advantages. PEI is widely recognized for its ability to interact with negatively charged cellular membranes, facilitating cellular uptake and enabling endosomal escape of the nanoparticles. Additionally, PEI contributes to the structural stability and biocompatibility of MSNs, making them more efficient carriers for therapeutic agents [42,43,44]. When combined with other functionalization, such as folic acid and organotin compounds, PEI-coated MSNs represent a potential breakthrough in cancer treatment. This approach not only reduces the undesirable side effects associated with conventional therapies but also allows for a decrease in the required dosage, enhancing patient safety and comfort. These combined functionalities pave the way for more effective and targeted therapeutic solutions, positioning PEI-modified MSNs as a promising alternative to traditional cancer treatments [45,46].

All the modifications mentioned above are carried out both on the internal and external structure of the nanoparticle. Furthermore, all are performed in a non-classical manner, creating covalent bonds between the MSN structure and the target molecule through ligands (TEDTH) [47,48]. The concept of non-classical systems refers to nanomaterials that function as a single therapeutic entity, without requiring the release of the active compound. In these systems, the functional molecules are covalently anchored to the nanoparticle surface, forming a stable hybrid structure in which the biological activity arises from the material as a whole. This approach contrasts with classical drug delivery systems, where the therapeutic effect depends on the diffusion and release of the encapsulated drug.

In addition, a distinctive aspect of this work is the incorporation of polyethyleneimine (PEI) as a multifunctional coating in the design of the nanocarriers. The presence of PEI offers several advantages that distinguish this study from previous approaches. On the one hand, PEI improves the colloidal stability of the nanoparticles and enables modulation of their surface charge, which plays a crucial role in their interaction with biological membranes and subsequent cellular internalization. On the other hand, the abundance of amine groups (–NH_2_) on the PEI chains provides reactive sites that remain available for covalent attachment of targeting ligands or therapeutic molecules. This dual functionality, enhancing both physicochemical stability and surface versatility, confers the system a high degree of tunability, positioning it as a promising platform for the development of more efficient and selective anticancer therapies.

Thus, this study focuses on the development and characterization of MSNs with advanced functionalization strategies aimed at the treatment of breast cancer. Two synthetic routes for incorporating organotin compounds into MSNs will be explored, examining their impact on the physicochemical properties and biological performance of the nanoparticles. In addition, the effects of functionalization with organotin(IV) complex (Sn), FA and PEI coating will be evaluated to optimize therapeutic efficacy, active targeting and biocompatibility. Through these approaches, this research aims to contribute to the development of next-generation nanoparticle systems for cancer therapy, providing innovative solutions to overcome current therapeutic challenges [49].

## 2. Materials and Methods

### 2.1. Synthesis and Characterization of Materials

#### 2.1.1. General Condition on the Synthesis and Characterization of the Materials

Reagents, including tetraethyl orthosilicate (TEOS), N-(3-Dimethylaminopropyl)-N′-ethylcarbodiimide hydrochloride (EDAC), N-Hydroxysuccinimide (NHS), folic acid (FA), 3-Mercaptopropionic Acid (MPP), triphenyltin(IV) chloride (Sn), and (N-morpholino) ethanesulfonic acid (MES), were obtained from Sigma Aldrich (St. Louis, MO, USA). Hexadecyltrimethylammonium bromide (CTAB) and N-[(3-Trimethoxysilyl)propyl]ethylenediamine triacetic acid trisodium salt (TEDTH) were sourced from Fluorochem (Glossop, Derbyshire, UK),while branched polyethyleneimine (PEI) and Traut’s reagent (TR) were purchased from Acros Organics (Geel, Belgium).

All reactions conducted under an inert atmosphere and dry nitrogen were carried out in standard Schlenk tubes. The solvents used in these reactions were purified through distillation using appropriate drying agents and degassed prior to use.

Solid-state UV-vis spectra were obtained using a Perkin Elmer (Waltham, MA, USA) LAMBDA 850+ UV/Vis Spectrophotometer. FT-IR vibrational spectra were recorded using KBr pellets with the material embedded inside, measured on a Spectrum Two™ 6700 FT-IR spectrophotometer (Perkin Elmer (Waltham, MA, USA)). Thermogravimetric analysis (TGA) for mass loss was conducted in the 30–800 °C range, employing a heating rate of 20 °C/min and a nitrogen flow of 50 mL/min, using a DSC/TGA Discovery SDT650 (New Castle, DE, USA). Nitrogen adsorption-desorption isotherms (BET) were measured with a Micromeritics ASAP 2020 porosimeter (Norcross, GA, USA). For X-ray diffraction (XRD) analysis, a Philips PW3040/00 X’Pert MPD/MRD diffractometer (Almelo, Overijssel, Netherlands) was employed, operating at 45 kV and 40 mA with a Cu Kα radiation source (λ = 1.5418 Å). Transmission electron microscopy (TEM) images were captured using a JEOL JEM 1010 instrument (Akishima, Tokyo, Japan) operating at 100 kV. Field emission scanning electron microscopy (FEG-SEM) images were captured using Apreo ChemiSEM S LoVac instrument (Waltham, MA, USA). Inductively coupled plasma (ICP) analysis for metal quantification was carried out on a Varian Vista AX Pro instrument (Palo Alto, CA, USA) (λSn = 235.485 nm).

Z-potential and Dynamic Light Scattering (DLS) measurements were performed in dispersion using the Litesizer 500 (Anton Paar (Graz, Styria, Austria)). Finally, cell activity assays for the materials were evaluated using an SP-2000UV Spectrophotometer (Ningbo, Zhejiang, China).

#### 2.1.2. Synthesis of MSN

Silica nanoparticles, used as carrier nanostructures, are prepared using the sol-gel process [50]. First, a solution of hexadecyltrimethylammonium bromide (CTAB, 1.00 g, 2.74 mmol) is prepared in Milli-Q water (480 mL) in a round-bottom flask with magnetic stirring to initiate the formation of micelles. Then, 3.50 mL of NaOH (2 M) is added to create a slightly basic environment. The mixture is then gradually heated to 80 °C. Once the target temperature is reached, tetraethyl orthosilicate (TEOS, 5.00 mL, 22.40 mmol) is added drop by drop to coat the micelles with silica and form the structure. The reaction is maintained for 2 h at this temperature with stirring. The resulting product is filtered and thoroughly washed with Milli-Q water (2 × 250 mL) and methanol (1 × 250 mL) to obtain a white solid. At this stage, the nanoparticles still do not have accessible pores for functionalization. To open the pores, the solid is subjected to calcination at 550 °C for 24 h.

#### 2.1.3. Activation of the MSN and Functionalization with the TEDTH Ligand. Preparation of MSN-TEDTH

To perform any functionalization on an empty MSN, it is necessary to remove any absorbed water and activate the silanol groups present in its structure. This is achieved by subjecting the material to vacuum at 90 °C for 24 h. After this time, the MSN (1.2 g) is dispersed in dry ethanol (30 mL) under an inert atmosphere, and the trisodium salt of the ligand N-[(3-Trimethoxysilyl)propyl]ethylenediamine triacetic acid trisodium salt (TEDTH, 952 µL, 2.59 mmol) is added. The mixture is allowed to react at 75 °C for 48 h under stirring. To obtain the solid product, the resulting mixture is centrifuged and washed with ethanol (2 × 20 mL) and diethyl ether (2 × 20 mL), then dried at 75 °C overnight.

#### 2.1.4. Formulation with PEI. Preparation of MSN-TEDTH-PEI

The coating with the polymer is achieved through an EDAC coupling between the amines of polyethyleneimine and the free carboxyl groups of TEDTH. To accomplish this, the protocol previously described by R. Badihi et al. [46] was followed. The precursor material (MSN-TEDTH, 1 g) is dispersed in MES buffer (0.1 M, 250 mL) with 1-ethyl-3-(3-dimethylaminopropyl)carbodiimide (EDAC, 665 mg, 3.47 mmol) and N-hydroxysuccinimide (NHS, 665 mg, 5.78 mmol), and the mixture is stirred for 1 h at room temperature. After this period, polyethyleneimine (PEI, 200 mg, 3.32 mmol) is added to the mixture, allowing the coating to proceed for 24 h. To obtain the final solid, the sample is centrifuged, washed with methanol (2 × 20 mL), and dried under vacuum at room temperature to avoid damaging the polymer.

#### 2.1.5. Functionalization with Folic Acid. Preparation of MSN-TEDTH-PEI-FA

Functionalization is carried out through the EDAC reaction described in the previous section, with variations in the quantities and reaction times. First, folic acid (FA, 160 mg, 0.36 mmol) was dispersed in dimethyl sulfoxide (DMSO, 20 mL), ensuring complete dispersion using ultrasound. This solution was then added to the previously prepared buffer (MES, 250 mL), along with 1-ethyl-3-(3-dimethylaminopropyl)carbodiimide (EDAC, 106.04 mg, 0.56 mmol) and N-hydroxysuccinimide (NHS, 106.04 mg, 0.92 mmol) and allowed to react for 15 min at room temperature with vigorous magnetic stirring. After this time, previously synthesized material (MSN-TEDTH-PEI, 800 mg) was incorporated, and the reaction was allowed to continue under the same conditions for 2 h. The resulting mixture was centrifuged, washed with ethanol (2 × 30 mL), and finally dried under vacuum at room temperature.

#### 2.1.6. Functionalization with Ligands MPP and TR. Preparation of MSN-TEDTH-PEI-FA-MPP and MSN-TEDTH-PEI-FA-TR

Functionalization with the ligand 3-Mercaptopropionic Acid (MPP) was carried out via an EDAC-type reaction between the amino groups present in PEI and FA and the carboxyl group present in the ligand, thereby leaving a free binding site through a thiol group. For this purpose, a solution of 3-Mercaptopropionic Acid (MPP, 49.2 µL, 0.56 mmol) in buffer (MES, 80 mL) was prepared, to which 1-ethyl-3-(3-dimethylaminopropyl)carbodiimide (EDAC, 162.55 mg, 0.85 mmol) and N-hydroxysuccinimide (NHS, 162.65 mg, 1.41 mmol) were added, allowing the reaction to proceed for 15 min under magnetic stirring at room temperature. After this time, the previously synthesized material (MSN-TEDTH-PEI-FA, 300 mg) was added to the solution and left to react for 24 h under the same conditions. Finally, the material was centrifuged, washed with ethanol (2 × 30 mL), and dried under vacuum at room temperature.

Functionalization with the second ligand was carried out following the protocol previously described by J. Dembélé [51], which is briefly outlined below. This reaction takes place on the primary amines of FA and PEI, creating binding sites through thiol groups.

In a Schlenk flask, a dispersion of the previously synthesized material (MSN-TEDTH-PEI-FA, 300 mg) was prepared in phosphate-buffered saline (PBS, 25.61 mL) and reacted with Traut’s reagent (TR, 116.63 mg, 0.85 mmol), also pre-dispersed in phosphate-buffered saline (PBS, 5.91 mL). The dispersion was subjected to a nitrogen atmosphere for 5 min, then left to react for 24 h at room temperature with vigorous magnetic stirring. After this time, the material was centrifuged and washed with PBS (2 × 30 mL) and ethanol (1 × 30 mL) to remove any potential salts formed. Finally, it was dried under vacuum at room temperature.

#### 2.1.7. Functionalization with Triphenyltin(IV) Chloride. Preparation of MSN-TEDTH-PEI-FA-MPP-Sn and MSN-TEDTH-PEI-FA-TR-Sn

The procedure for both materials is the same, utilizing the free SH groups of the ligands to bind a Sn compound with a Cl in its structure. The protocol described previously by D. Díaz-García [36] was followed and is briefly outlined below.

The reactions were conducted under an inert atmosphere, achieved by performing vacuum/nitrogen cycles in a Schlenk flask containing the previously synthesized material (MSN-TEDTH-PEI-FA-MPP or MSN-TEDTH-PEI-FA-TR, 150 mg) and triphenyltin(IV) chloride (SnPh_3_Cl, 48.71 mg, 0.13 mmol), which was subsequently dispersed in dry toluene (15 mL). After dispersing the mixture with magnetic stirring, triethylamine (NEt_3_, 36.26 µL, 0.26 mmol) was added, and the reaction was allowed to proceed for 48 h at 55 °C to prevent polymer degradation. After this period, the material was centrifuged and washed with toluene (2 × 30 mL) and ethanol (1 × 30 mL), then dried under vacuum at room temperature.

#### 2.1.8. Metal Release Studies (ICP-AES)

Although the materials are functionalized in a non-classical manner and it is expected that there will be no release of the metal into the environment, its behavior in a simulated biological medium is studied. For this purpose, dispersions of the final materials (MSN-TEDTH-PEI-FA-MPP-Sn and MSN-TEDTH-PEI-FA-TR-Sn) are prepared in PBS pH = 7.4 at a concentration of 1 mg/mL and incubated for 8 and 72 h under orbital agitation at 37 °C. After this time, the dispersion is centrifuged, and the supernatant is filtered. Finally, the amount of metal released into the medium is measured using ICP-AES.

#### 2.1.9. Experimental Details on Z-Potential and Dynamic Light Scattering (DLS)

Final materials were characterized using Z-potential to quantify their surface charge and assess their behavior in biological environments. For this, dispersions of the materials (MSN-TEDTH-PEI-FA-MPP-Sn and MSN-TEDTH-PEI-FA-TR-Sn) were prepared in saline medium (PBS, pH = 7.4) at a concentration of 1 mg/mL and sonicated for 2 min. The dispersion was then transferred to the measurement cell using a syringe, and the quantification was performed. Additionally, both Z-potential and DLS measurements were carried out in the cell culture medium to evaluate the colloidal stability and hydrodynamic behavior of the nanoparticles under the same conditions used in the biological assays.

### 2.2. Antitumoral Activity of the Synthesized Materials

#### 2.2.1. Cell Line and Cell Culture Conditions

Cell lines used were Hek 293T as the healthy line and MCF-7 as the breast cancer tumor line. Both were cultured in DMEM (Corning^®^ GlutaGro™ (Corning, NY, USA)) medium supplemented with 10% Fetal Bovine Serum (FBS, Cytiva, 0.1 µM) and 1% antibiotics (Penicillin-Streptomycin, Corning^®^). The medium was renewed approximately every 48 to 72 h, using Trypsin (Corning^®^) to detach the cells. Both cell lines were incubated at 37 °C with 5% CO_2_. The human embryonic kidney cell line Hek 293T and the human breast adenocarcinoma cell line MCF-7 were obtained from the American Type Culture Collection (ATCC, Manassas, VA, USA). Cells were cultured and maintained according to the supplier’s recommendations.

#### 2.2.2. MTT Cytotoxicity Assay

To perform this assay, 5000 cells per well are seeded on a 96-well plate on the first day. After 48 h, the culture medium is replaced with medium containing dispersions of the materials. A stock dispersion of 2 mg/mL in H_2_O is prepared using sonication, followed by dilutions in the culture medium within a range of 10 to 500 µM. After 24 h, MTT reagent (1 mg/mL in culture medium) is added in a 1:0.1 ratio and incubated for 3 h to allow the formation of formazan salts. Subsequently, the medium is discarded and replaced with DMSO, which breaks the cell membranes with the help of agitation, allowing the release of salt to be measured. Finally, absorbance is measured at 570 nm using a plate reader.

## 3. Results and Discussion

### 3.1. Synthesis, Functionalization and Characterization of the Studied Nanostructured Materials

**Scheme 1:**
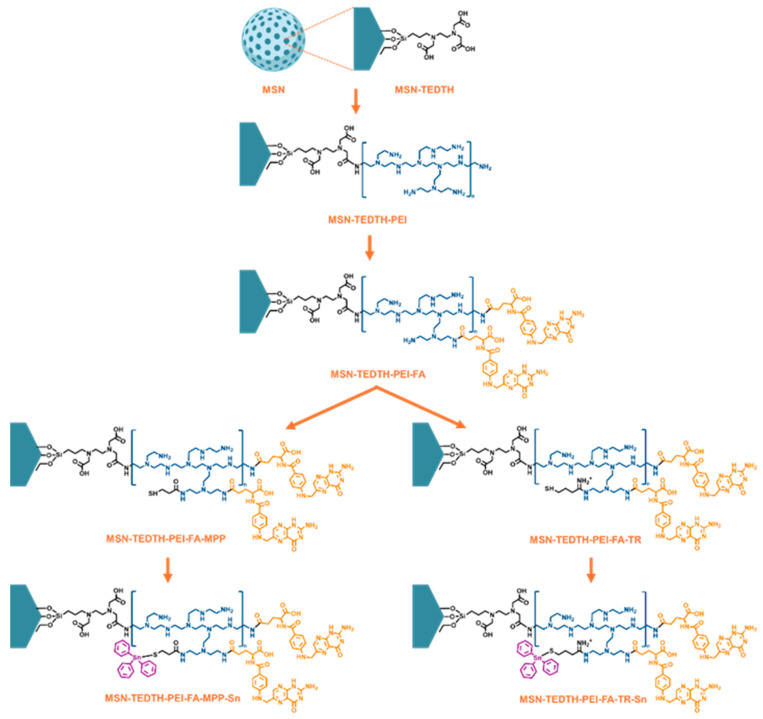
Scheme of functionalization of MSN with the different ligands and compounds of interest.

All the functionalization carried out in this work are performed on a mesoporous silica nanoparticle platform (MSN), synthesized using the sol-gel method. Once the nanoparticle is obtained, binding points are created using carboxyl groups with the ligand N-[(3-trimethoxysilyl)propyl]ethylenediaminetriacetic acid (TEDTH). The formulation is then carried out with the polymer polyethyleneimine (PEI) via an EDAC-type reaction. PEI possesses amino groups in its structure, which also serve as binding points. The next step involves functionalization with the active targeting compound folic acid (FA), again using an EDAC reaction, which covalently binds to the primary amines of the polymer. Subsequently, the system is functionalized with two different ligands: mercaptopropionic acid (MPP) and Traut’s reagent (TR), which bind to the primary amines of PEI and FA, with the latter being the most available. Finally, a tin(IV) fragment is incorporated to provide the therapeutic component, binding to the free thiol groups of the previously introduced ligands (Scheme 1). A thorough physicochemical characterization of the materials was subsequently performed, followed by the evaluation of their potential as antitumor agents in breast cancer through cell viability studies.

#### 3.1.1. UV–Visible Spectroscopy Studies (DR UV-Vis)

**Figure 1:**
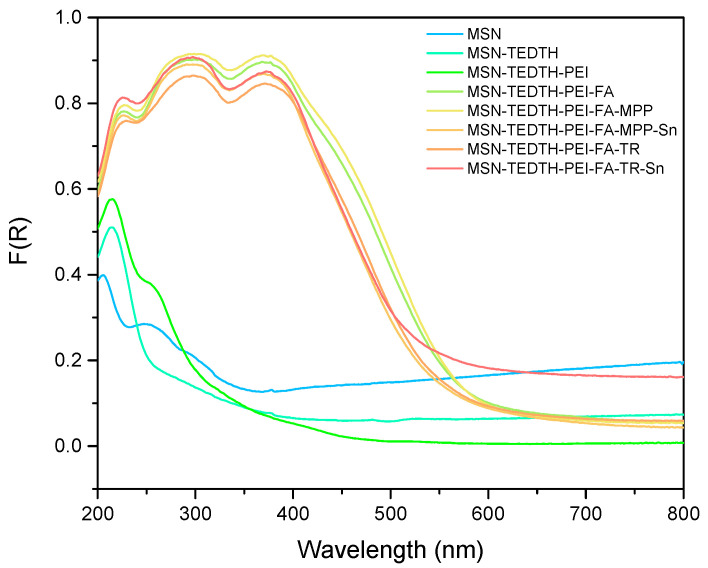
UV-vis spectra from the measurement of all the synthesized materials, showing the maximum absorption peaks of the compounds.

Figure 1 shows the results obtained by measuring all the synthesized and functionalized materials using the UV-vis spectrophotometer. It illustrates the absorption maxima of all the compounds used to derivatize the starting silica.

The starting material does not show any significant absorption peak, apart from an increase in intensity at very low wavelengths. However, after functionalization with the TEDTH ligand, a peak appears around 210 nm. The appearance of this new peak is to be expected after ligand functionalization, since compounds containing double bonds such as the C=O group present in the TEDTH ligand usually absorb in this region. Compounds with carboxyl or nitrogen-containing groups also exhibit absorptions in this part of the spectrum [52].

Subsequently, all materials formulated with PEI show a new absorption peak at 260 nm, which increases in intensity as the material is further derivatized with other compounds such as folic acid, ligands, and the Sn complex. The appearance of this peak upon PEI functionalization is attributed to the amino groups present in the polymer structure [53]. In addition, materials containing FA exhibit two clear absorption peaks at 320 and 390 nm, as reported in previous studies [35]. The first peak is associated with the aromatic rings of the molecule, while the second corresponds to the amino and carboxylic functional groups.

Finally, when the materials contain the organotin(IV) compound, an absorption peak is expected around 220 nm, due to the aromatic rings present in the metal complex [54]. However, it is difficult to observe it properly due to the overlap with the ligand’s peak in a nearby region, which is present in all the compounds. However, a visible increase in intensity of the signal can be observed.

To facilitate the interpretation of the UV–vis spectra and better visualize the characteristic absorption bands associated with the different functional groups, magnified views of the most relevant spectral regions have been included in the Supplementary Material (Figure S1). These enlarged images, corresponding to the ranges of 200–450 nm and 300–550 nm, allow clearer observation of the absorption signals related to the functionalization of the nanoparticles.

#### 3.1.2. FT-IR Studies

Another characterization technique that can be used to confirm the correct functionalization of MSN is FT-IR (Fourier-transform infrared spectroscopy). This technique allows the observation of new bands corresponding to the vibrations of the new bonds formed between the ligands and compounds introduced, as the functionalization is carried out by covalent binding.

**Figure 2:**
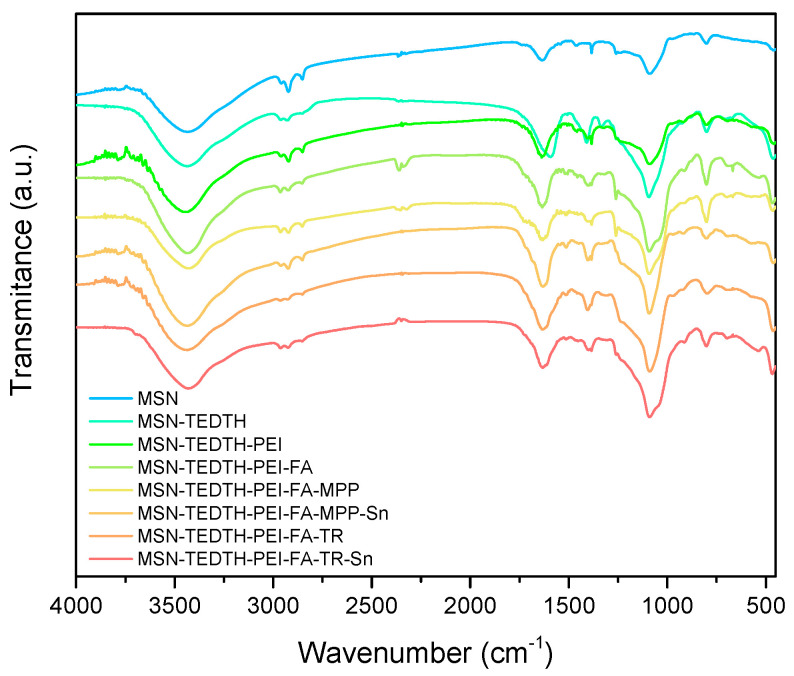
FT-IR spectra of all the synthesized materials, showing all the bands belonging to the binding vibrations of the compounds of interest.

Figure 2 shows the different spectra obtained. In the case of the starting nanomaterial (MSN), several characteristic bands can be seen. The first of these appears in the range of 3400–3500 cm^−1^ and corresponds to the O-H bonds of the silanol groups (Si-OH) in the silica structure or to any water that may have been absorbed in the material. Other characteristic vibration bands of the starting material are seen at 1100 cm^−1^ due to the stretching of Si-O-Si bonds, and at 800 cm^−1^, corresponding again to Si-OH presence. When the material is functionalized with TEDTH, a new band appears at 3000 cm^−1^ typical of the C-H stretching vibrations of the alkane groups of the ligand. A band at 1650 cm^−1^ due to the C=O bonds of the carboxyl groups was also observed [55]. Upon formulation with PEI, new bands appear at 3440 cm^−1^ and 1000–1200 cm^−1^, corresponding to free amine groups and the stretching of -CONH_2_ bonds [56]. The band at 2850 cm^−1^ also appears in the PEI functionalization and intensifies with FA functionalization, as it is associated with the N-H bond present in both compounds [31,57]. Finally, when the materials are functionalized with the metal compound, a new band appears at 750 cm^−1^, which is more difficult to observe due to its proximity to previous bands but corresponds to the Sn bond vibration [31,35,37,54,57].

The appearance of all these bands in the FT-IR spectrum indicates that the functionalization with all the compounds of interest has been successful.

To facilitate the interpretation of the FT-IR spectra, magnified views of the most relevant regions have been included in the Supplementary Material (Figure S2). These enlarged images correspond to the ranges of 4000–2500 cm^−1^ and 1800–400 cm^−1^ and include the assignment of the main absorption bands with their respective wavenumbers and functional groups. This representation allows a clearer identification of the characteristic vibrations associated with the different functionalization steps of the nanoparticles.

#### 3.1.3. Quantification of Interesting Molecules by TG, ICP and XFR

**Figure 3:**
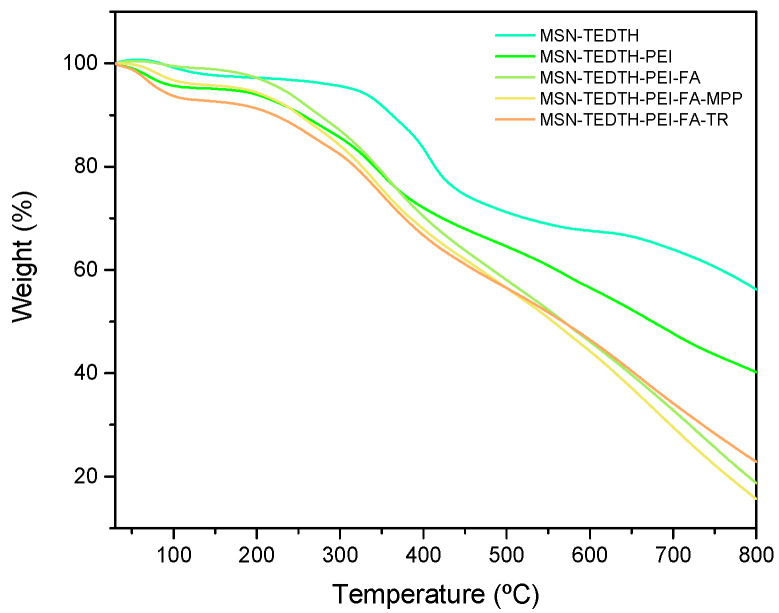
TG plot obtained by measuring TEDTH, PEI, FA, MPP and TR functionalized materials from 30 to 800 °C.

It is not only important to confirm that the functionalization has been carried out correctly, but also to quantify the amount of ligand (TEDTH), polymer (PEI), and active targeting compound (FA) present in the material. To do this, thermogravimetric analysis (TG) was carried out (Figure 3 and Table 1). It was determined that the functionalization with the ligand is 31.89% and 0.74 mmol/g. The theoretical amount for the functionalization was 1:1, and previous studies [54] have shown that this amount of ligand is sufficient to create the necessary binding sites to continue attaching the other molecules of interest. Therefore, the incorporation of approximately one third of the theoretical amount of the ligand seems to be adequate for the purposes of this work. The amount of PEI that binds to the material is 11.12% and 2.64 mmol/g, compared to the theoretical ratio of 1:0.2 introduced. Although this amount is just over half of the theoretical value, it is enough to enhance the diversity of the nanoparticle, which is the goal of this work, as well as to create new binding sites for the remaining molecules of interest via the numerous primary amines in PEI. In previously published studies, it has been observed that the mass loss was 7.9%, so the value obtained is similar to what has been previously reported and is likely to be sufficient to demonstrate its activity for biological applications [58]. It is important to note that regardless of the actual amount of polymer present on the nanoparticle, the number of free amino groups on its surface will be very high. Since it is a branched polymer, it provides a large number of primary amines, and therefore, the number of anchoring points for future ligands and molecules of interest will also be high. Finally, the functionalization with FA is 16.46% and 0.39 mmol/g, which is quite close to the theoretical value of 20%, indicating that the functionalization with TEDTH and PEI has proceeded correctly. As mentioned previously, the functionalization with the ligand and the polymer was highly effective, generating numerous anchoring points for molecules such as folic acid. In this work, a real loading value very close to the theoretical one was achieved, thanks to the presence of PEI which has a high number of amino groups. In previous studies carried out by the group, the amount of FA incorporated into the nanoparticle ranged from 0.17% to 2.4% [31,57]. Therefore, it can be stated that, in this work, up to 15 times more of this molecule of interest was successfully incorporated due to the PEI functionalization. It can also be concluded that the role of PEI is not only to improve the dispersibility of the material, but also to generate a greater number of amino binding sites for subsequent reactions, which has been demonstrated to be effective. The observed differences between the two ligands are remarkable. The MSN-TEDTH-PEI-FA-TR material exhibits significantly higher ligand loading (6.85%) compared to MSN-TEDTH-PEI-FA-MPP (0.54%). This variation indicates a stronger interaction and higher affinity of the TR ligand for the modified surface, which can be attributed to its chemical structure and the presence of additional coordination sites. Consequently, this enhanced anchoring capacity directly influences the subsequent incorporation of the metallic center, favoring a more efficient complexation and higher metal content in the final material.

Once the ligands and molecules of interest have been quantified, it is important to determine the amount of therapeutic agent present in each of the final nanomaterials. For this purpose, ICP-AES was used, as it provides the metal content in the material, which is crucial for future *in vitro* assays.

Material digestions were carried out at a concentration of 1 mg/mL in 2 M KOH, and the samples were stirred at room temperature for 48 h. After this time, the supernatant was filtered and measured. The value obtained (Table 1) for the material MSN-TEDTH-PEI-FA-MPP-Sn was 0.314 ± 0.001%, while for the material MSN-TEDTH-PEI-FA-TR-Sn, it was 0.526 ± 0.001% Sn. These are similar values, although it is worth noting that the incorporation of metal anchored through Traut’s reagent is 1.7 times greater, making it more effective. Although both values differ from the theoretical amount, previous studies, including the present one, have shown that this quantity is more than sufficient for the material to exhibit activity as an antitumor agent [35,37]. In the last column of Table 1, the elemental values obtained by XRF are shown. This technique was performed to verify how the metal was incorporated into the nanoparticle. In the case of the material functionalized with MPP, the amount of Cl is 8.7 times higher than that of S, indicating that some part of the metal complex might also be adsorbed onto the nanoparticle surface, leaving chlorine free within its structure. In contrast, for the material in which the metal complex was incorporated via TR, the amounts of both elements are similar. This suggests that in this case, the metal complex likely formed predominantly through covalent bonding, results in the elimination of the chlorine from the reagent.

#### 3.1.4. Nitrogen Adsorption-Desorption Isotherms (BET)

**Figure 4:**
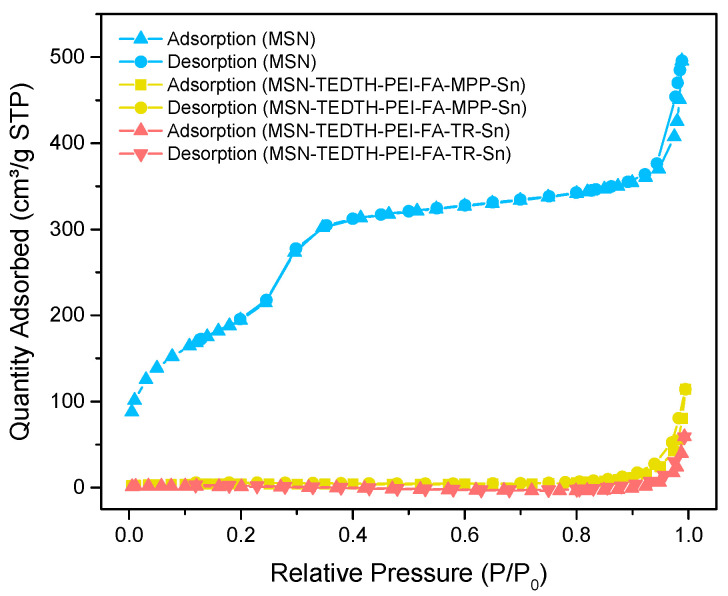
Nitrogen desorption-adsorption isotherms of materials: (blue) MSN; (yellow) MSN-TEDTH-PEI-FA-MPP-Sn; (orange) MSN-TEDTH-PEI-FA-TR-Sn.

This technique provides information about the type of porosity of the material, offering quantitative data on the surface area, pore volume, and pore diameter of the nanoparticle. It also informs us about whether the functionalization has been carried out correctly and where it occurred, providing information about the type of isotherm obtained (Figure 4 and Figure S3 of Supplementary Material).

Thus, the starting material (MSN) presents type IV isotherms according to IUPAC [58]. These are characteristics of mesoporous solids, indicating that the synthesis of the starting nanoplatforms was carried out correctly. Additionally, a hysteresis loop is observed, which occurs due to an increase in the amount of N_2_ absorbed by the material, that is, due to the condensation of the gas in the pores of the adsorbent and the increased interaction, leading to an H2 hysteresis loop. This suggests strong interaction between the adsorbate and the adsorbent. Initially, the pores of the nanomaterial fill in a monolayer fashion, with no apparent hindrance, until the relative pressure is reached, and the material starts filling in multilayers progressively and more slowly. Once the relative pressure reaches 1, the gas condensation occurs, followed by its desorption from the pores as the pressure decreases.

The final materials (MSN-TEDTH-PEI-FA-MPP-Sn and MSN-TEDTH-PEI-FA-TR-Sn) present isotherms which are close to the shape of type II isotherm according to IUPAC. This type of isotherm is characteristic of macroporous or non-porous solids. This indicates that the nanoparticle is highly functionalized, resulting in weak interactions between N_2_ and the material, which prevents the typical isotherm characteristics of the initial material from being maintained.

The starting MSN material has a surface area of 713.24 m^2^/g and a pore volume of 0.63 cm^3^/g. The final materials show a significant reduction in both parameters. For MSN-TEDTH-PEI-FA-MPP-Sn, the surface area is 14.21 m^2^/g and the pore volume is 0.06 cm^3^/g, while for MSN-TEDTH-PEI-FA-TR-Sn, the surface area is 3.21 m^2^/g and the pore volume is 0.03 cm^3^/g. These high reductions are due to the successful functionalization with relatively high amounts of the compounds of interest. Among the functionalization steps, the incorporation of PEI, a large molecule that saturates the surface of the nanoparticle, plays a key role because it also offers advantages such as new binding sites for subsequent functionalization reactions and increases the nanoparticle’s biodiversity.

In previous studies, it has been observed that the loss of BET surface area associated with the functionalization of MSN with PEI can reach up to 820 m^2^/g when the polymer is present in high amounts. Therefore, a BET surface area loss of up to 710 m^2^/g falls within the expected range and indicates that the incorporation of both the polymer and the target molecules seems to be successfully achieved [59]. Notably, this technique also informs us that functionalization has not only occurred on the external surface of the material but also within the internal pores, which is why the pore volume also decreases significantly. When comparing the pore volume of the starting material and the final material, a decrease of up to 0.6 cm^3^/g can be observed, indicating that the functionalization has taken place within the pores of the nanoparticle. Furthermore, when comparing the values obtained for the material functionalized with the MPP ligand and with the TR ligand, it can be seen that the latter has a pore volume reduced by half. This may suggest that a higher degree of functionalization occurred in the MSN-TEDTH-PEI-FA-TR-Sn material. As observed in the ICP analysis, the amount of incorporated metal was higher, which is probably related, as mentioned, to the decrease in pore volume.

#### 3.1.5. Powder X-Ray Diffraction Studies (XRD)

Another technique that provides detailed information about the structure of nanomaterials and potential changes resulting from functionalization is powder X-ray diffraction (XRD). This technique produces a unique diffractogram for each material, as the diffraction peaks reflect the specific internal structure and allow for quantitative data on the diffraction planes.

**Figure 5:**
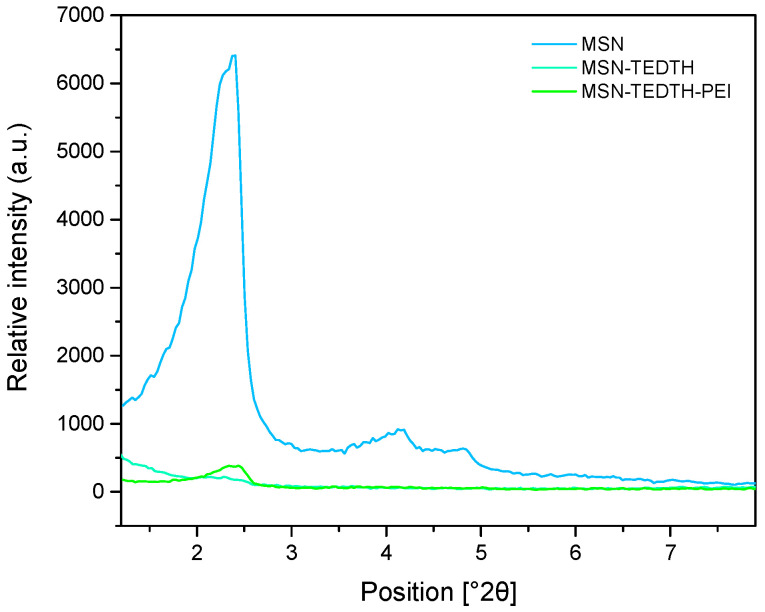
XRD spectra obtained by measuring the selected materials in powder form.

In Figure 5, the diffraction spectra of the materials, MSN, MSN-TEDTH, and MSN-TEDTH-PEI, are shown. For the starting material, the three characteristic peaks corresponding to the Miller planes [100], [110], and [200] are clearly visible, confirming the ordered mesoporous structure observed at low angles. The values for each plane can be seen in Table 2, the appearance of three peaks confirms that the pores in these materials are arranged in a hexagonal structure as previously found in the literature [60].

Furthermore, as shown in Figure 5 and Table 2, when the starting material is functionalized with the TEDTH ligand, the intensity of the peaks corresponding to these planes significantly decreases, and in some cases, the signals attributed with the mesoscopic order almost disappear, indicating almost a complete saturation of the pores confirming that the functionalization has been successful, as the ligand has entered the pores, altering the diffraction pattern since the beam interacts differently compared to when the pores were empty.

A similar observation is made for the material functionalized with PEI; given that PEI is a large molecule, it anchors to the silica as a formulation, obstructing the clear visibility of the previously mentioned planes. In this case, only the [100] plane is observable, with a slight shift and very low relative intensity. This behavior has been already observed in previously published materials, since in nanoparticles containing PEI, no peaks appear in XRD at low angle associated with the polymer [61,62].

#### 3.1.6. Electronic Microscopy Studies (TEM)

In addition to confirm the correct functionalization of the starting nanomaterial and studying its porosity, it is necessary to verify the materials’ size and morphology. For this purpose, transmission electron microscopy (TEM) images were taken, processed, and analyzed using the ImageJ software 1.53e [63].

**Figure 6:**
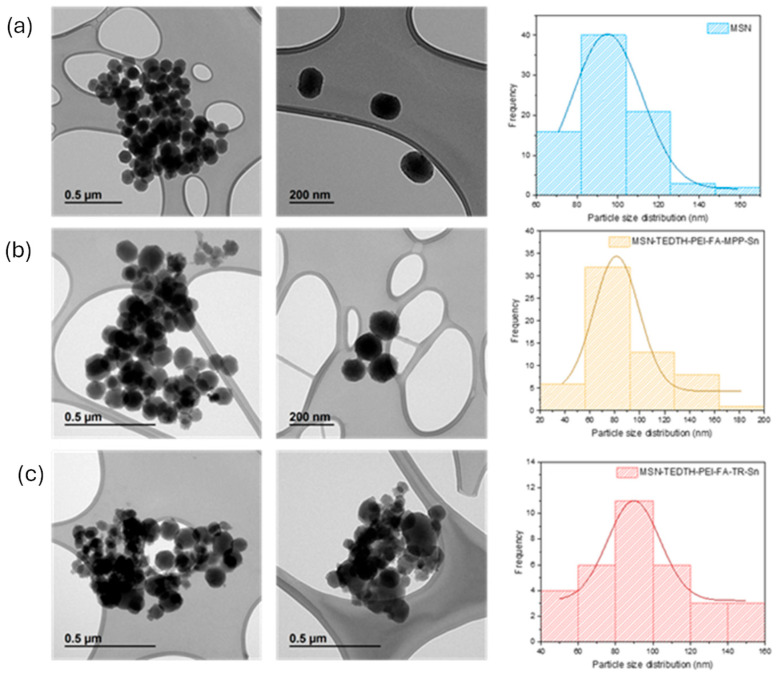
TEM images and Gaussian particle size distribution of the materials: (**a**) MSN; (**b**) MSN-TEDTH-PEI-FA-MPP-Sn and (**c**) MSN-TEDTH-PEI-FA-TR-Sn. Histograms were plotted with the measurement of a minimum of 30 nanoparticles.

Figure 6 shows the images obtained for both the starting material and the final materials. Several observations can be summarized, all materials exhibit a quasi-spherical morphology, as expected. In some of the images where the materials are observed more closely, it is even possible to see their porosity (Figure S4), which corroborates the high surface area what was previously observed in the BET analysis. Secondly, the final materials maintain their morphology and structure, which means that the functionalization with the different molecules of interest and exposure to different reactions have not affected their structure, which is highly advantageous. Thirdly, it can be observed that the samples are quite homogeneous, as the nanoparticles are very similar to each other.

In Figure 6, the histograms with the Gaussian pore size distribution obtained by measuring all the nanoparticles can be seen. It was determined that the average size of the MSN nanoparticles is 97.92 ± 19.70 nm, while for the final materials, MSN-TEDTH-PEI-FA-MPP-Sn and MSN-TEDTH-PEI-FA-TR-Sn, the sizes are 99.39 ± 21.82 nm and 103.13 ± 33.34 nm, respectively. No significant variation in nanoparticle size is observed between the starting material and the final products. This indicates that the functionalization with ligands and other molecules of interest does not alter the nanoparticles, either in terms of morphology or size, and no degradation or alterations of the MSNs are detected after derivatization.

#### 3.1.7. Field Emission Scanning Electron Microscopy (FEG-SEM)

To further confirm the successful functionalization of the mesoporous silica nanoparticles, elemental mapping analysis was performed by field emission scanning electron microscopy (FEG-SEM) (Figures S5 and S6, Tables S1 and S2). The mapping images revealed the presence of O, Si, Sn, and N elements in the nanomaterial. These elements were homogeneously distributed throughout the nanoparticles, indicating a uniform incorporation of the organotin species on the silica surface.

### 3.2. Studies in a Simulated Biological Environment

#### 3.2.1. Release Studies

**Figure 7:**
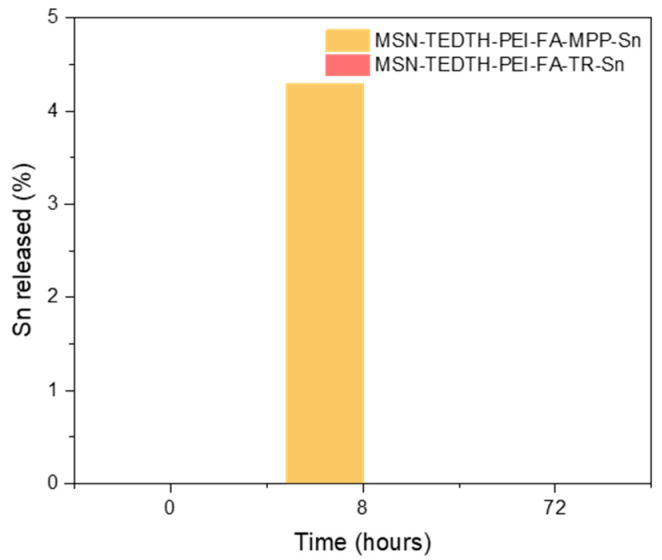
Graph of metal release in simulated biological media of the final materials MSN-TEDTH-PEI-FA-MPP-Sn and MSN-TEDTH-PEI-FA-TR-Sn.

The dispersions were prepared as described in the Materials and Methods section (Section 2). Once the metal release results were obtained by ICP-AES, as shown in Figure 7 and Table 3, the metal release was undetectable by the equipment in all measurements except for the material MSN-TEDTH-PEI-FA-MPP-Sn after 8 h, where a low release of 4.3% was observed relative to the total Sn content in the material.

Although the found tin amount at 8 h could suggest that the therapeutic agent is being released into the biological medium, after 72 h the amount of tin is not within the detection range of the ICP and it is possible that a small amount of poorly anchored metal complex was present in the measured sample being released into the medium and subsequently going to insoluble tin-containing species. A similar behavior has already been observed in previous studies reported by our group [37,54], where a very small amount of the same metal complex was released, for instance, at 4 h, but became negligible again by 24 h. In general, in previous studies the amount of metal released into the medium has always been below 1% for materials based on MSNs and containing the same metal complex. Although in this case the amount is slightly higher, when compared to the total metal content in the material (0.53%), the amount released into the medium is very small and does not validate a significant amount of adsorbed tin species and points towards a “non-classical” drug-delivery behavior for MSN-TEDTH-PEI-FA-MPP-Sn, as once again, this technique demonstrates that the metal complex is covalently anchored to the nanoparticle through the MPP and TR ligands, therefore, acting as a whole within the cells.

#### 3.2.2. Z-Potential and Dynamic Light Scattering (DLS)

Table 4 summarizes the zeta potential values of the final MSN-based materials measured in buffered saline and in simulated biological medium, prior to evaluating their potential antitumor activity *in vitro*. In PBS, the materials MSN-TEDTH-PEI-FA-MPP-Sn and MSN-TEDTH-PEI-FA-TR-Sn exhibit negative ζ-potential values in the range of −20 to −30 mV [64]. In previous studies carried out with MSN-based nanoparticles functionalized with the same metal complex but in the absence of PEI, ζ-potential values at similar pH were between −30 and −40 mV [35]. In the present case, the less negative values are consistent with the presence of PEI, whose amino groups contribute positive charges to the nanoparticle surface. This partial reduction in the negative surface charge is advantageous in a biological therapeutic context, as literature reports indicate that ζ-potential values in the approximate range between +20 and −30 mV favor efficient interaction of nanoparticles with cell membranes while maintaining sufficient colloidal stability [64]. Both systems fall within this window.

Furthermore, the negative Z-potential values indicate good colloidal stability due to electrostatic repulsion. This is beneficial as it may reduce the risk of nanoparticle aggregation and would help facilitating the appearance of undesired interactions when the material is in a complicated biological environment, such as the tumor microenvironment. In addition, the negative ζ-potential values in PBS suggest adequate electrostatic repulsion between particles, which should help minimize aggregation and reduce undesired nonspecific interactions when the materials are dispersed in complex biological environments such as the tumor microenvironment. A small difference of ca. 4 mV is observed between the MPP- and TR-functionalized systems, suggesting that the ligand used to anchor the organotin complex may slightly modulate the overall surface charge. This observation is in line with the formation of iminium-type species in the case of TR, which can partially compensate the negative charge of the silica surface more effectively than MPP.

To better approximate the conditions used in the *in vitro* experiments, ζ-potential values were also measured in the same complete cell culture medium employed for the biological assays (Table 4). Under these conditions, MSN-TEDTH-PEI-FA-MPP-Sn and MSN-TEDTH-PEI-FA-TR-Sn exhibited ζ-potentials of −8.61 mV and −8.11 mV, respectively. The marked decrease in the magnitude of the ζ-potential compared with PBS reflects the partial screening of surface charges by ions, proteins and other biomolecules present in the medium. Although the absolute values are closer to neutrality, they are still compatible with a reasonably stable dispersion over the timescale of the experiments, and they provide a realistic description of the surface properties of the systems under cell exposure conditions.

DLS measurements in biological medium (Table S3) revealed a similar hydrodynamic behavior for both systems. MSN-TEDTH-PEI-FA-TR-Sn nanoparticles displayed an average hydrodynamic diameter of approximately 200 nm, whereas MSN-TEDTH-PEI-FA-MPP-Sn showed sizes of around 140 nm. The presence of a predominant peak in this range, indicates that both formulations form relatively well-dispersed colloidal suspensions in cell culture medium. The larger hydrodynamic diameters compared with the core sizes observed by TEM are fully consistent with the contribution of the organic coating (PEI, FA and the organotin complex), a possible protein corona formed under biological conditions, and a limited degree of nanoparticle association in the medium. These features are expected to favor cellular interaction and uptake while preserving sufficient colloidal stability during the *in vitro* assays.

### 3.3. Biological Studies

#### MTT Assay

Toxicity and cytotoxicity studies of the synthesized materials were conducted to evaluate their potential antitumor activity against breast cancer cells. For this purpose, the cell lines Hek 293T and MCF-7 were used.

**Figure 8:**
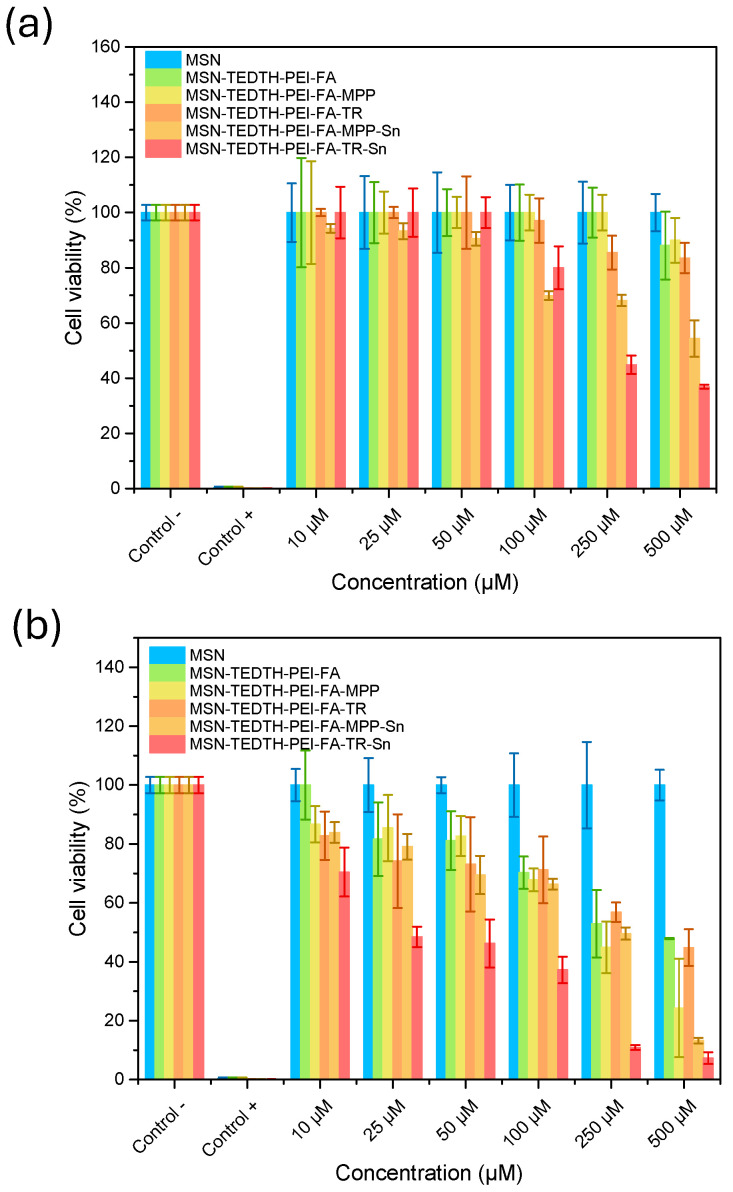
Percentage viability as a function of material concentration, obtained by incubating the materials in the cell lines: (**a**) Hek 293T and (**b**) MCF-7. Cells were seeded and grown for 48 h, followed by a 24 h exposure to the materials at 37 °C and 5% CO_2_. The negative control (Control –) consisted of cells without any added compound, while the positive control (Control +) contained 50% DMSO to evaluate maximal cytotoxicity.

Figure 8 illustrates the viability graphs, showing the percentage of cell viability as a function of the total material concentration. In both cell lines, it was observed that the starting material exhibits no toxicity, confirming that mesoporous silica nanoparticles (MSNs) serve as an excellent starting vehicle due to their biocompatibility [65]. Overall, all materials demonstrated low toxicity toward healthy cells while exhibiting cytotoxic activity against tumor cells. This is advantageous, as it indicates that the materials are unlikely to cause significant side effects due to their low action against healthy cells of organs where nanoparticles could accumulate before excretion.

These findings are corroborated by the results in Table 5, where all materials show IC_50_ values above 500 µM in terms of total material concentration. Only the MSN-TEDTH-PEI-FA-TR-Sn material shows some level of toxicity, but at a high concentration of 228.1 µM for the final material and 1.2 µM for the total Sn concentration. However, the actual administered dose would not reach these levels, because, as shown in Table 6, the same material requires only 23.97 µM of total material and 0.13 µM of Sn concentration to eliminate 50% of the tumor cell population. Therefore, with a dose up to 10 times lower, tumor cells can be eradicated without causing any harm to healthy cells. At concentrations close to 25 µM, the final material maintains 100% cell viability.

When comparing the IC_50_ values as a function of the metal with those obtained in previous studies by the group against breast cancer cells (IC_50_ = 4–19 µM) or ovarian cancer cells (IC_50_ = 1–5.5 µM) [35,37,66], it can be observed that the amount of metal required is up to 5.4 µM lower, which is attractive from the therapeutic perspective, as there is a small lowering of the dose.

Another important factor to consider when evaluating whether a final material has potential as an antitumor agent is that it should not exhibit activity against healthy cells. By comparing the values shown in Table 5 and Table 6, it can be observed that both final materials display primarily cytotoxic activity toward tumor cells, which is likely to be effective for future *in vivo* applications.

## 4. Conclusions

This work addresses the synthesis of mesoporous silica nanoparticles (MSNs) and their subsequent functionalization to develop compounds with potential interest as antitumor treatments for breast cancer. To achieve this, the following compounds were non-classically incorporated onto their surface: folic acid, as an agent capable of providing active targeting; polyethyleneimine, to enhance the material’s bioavailability; and an organotin(IV) compound, as the treatment agent, using two different molecules as linkers to the nanoparticle.

Once the nanomaterials were synthesized, their formation was verified using various physicochemical characterization techniques, which confirmed that the functionalization was successful and carried out non-classically on both the internal and external surfaces of the nanomaterial.

The combined ζ-potential and DLS analyses indicate that the materials retain adequate dispersion in saline buffer and in complete cell culture medium, maintaining hydrodynamic sizes and charge values within ranges typically associated with favorable cellular interactions. Overall, the results support the suitability of these functionalized MSNs as candidates for further biological evaluation. The observed behavior in complex media, together with the structural integrity of the systems, suggests that both formulations should operate robustly under the conditions relevant *in vitro* assays, which were performed using Hek 293T and MCF-7 cell lines, which demonstrated that all synthesized materials are non-toxic to healthy cells and that only the final materials containing the therapeutic agent exhibit cytotoxicity toward the cancer cell line. The most promising material for future *in vivo* studies is MSN-TEDTH-PEI-FA-TR-Sn, as it requires ten times less total material, and six times less therapeutic agent compared to the MPP-functionalized material to achieve 50% reduction in cell viability. Moreover, the amount of therapeutic agent required is up to 5 µM lower than that reported in previous studies with similar materials. Therefore, this material shows strong potential activity against the breast cancer cell line.

## Figures and Tables

**Table 1:** Values obtained from the quantification of the functionalized materials by TG, ICP-AES and XRF.

Materials	Weight (%)	mmol/g	Sn (%)	Other Elements (%)
MSN-TEDTH	31.89 (TEDTH)	0.74	-	-
MSN-TEDTH-PEI	11.12 (PEI)	2.64	-	-
MSN-TEDTH-PEI-FA	16.46 (FA)	0.39	-	-
MSN-TEDTH-PEI-FA-MPP	0.54 (MPP)	0.06		
MSN-TEDTH-PEI-FA-TR	6.85 (TR)	0.66		
MSN-TEDTH-PEI-FA-MPP-Sn	-	-	0.314 ± 0.001	3.56 (S) 30.96 (Cl)
MSN-TEDTH-PEI-FA-TR-Sn	-	-	0.526 ± 0.001	10.82 (S) 11.40 (Cl)

**Table 2:** XRD Miller plane data obtained for the selected materials.

Materials	(hkl)	2θ (°)	dhkl (Å)
MSN	100	2.41	36.57
	110	4.17	21.18
	200	4.84	18.25
MSN-TEDTH	100	2.31	-
MSN-TEDTH-PEI	100	2.47	35.67

**Table 3:** Metal release of MSN-TEDTH-PEI-FA-MPP-Sn and MSN-TEDTH-PEI-FA-TR-Sn measured by ICP-AES after incubation in PBS at 37 °C for 0, 8, and 72 h.

Materials	Release (%)
MSN-TEDTH-PEI-FA-MPP-Sn (0 h)	<0.1
MSN-TEDTH-PEI-FA-TR-Sn (0 h)	<0.1
MSN-TEDTH-PEI-FA-MPP-Sn (8 h)	4.3
MSN-TEDTH-PEI-FA-TR-Sn (8 h)	<0.1
MSN-TEDTH-PEI-FA-MPP-Sn (72 h)	<0.1
MSN-TEDTH-PEI-FA-TR-Sn (72 h)	<0.1

**Table 4:** Data obtained in the Z-potential averages of the final materials in saline (PBS) and in cell culture medium.

Materials	Z-Potential (mV)	Standard Deviation (mV)
MSN-TEDTH-PEI-FA-MPP-Sn	−25.821 ^a^	1.011
MSN-TEDTH-PEI-FA-TR-Sn	−21.918 ^a^	1.137
MSN-TEDTH-PEI-FA-MPP-Sn	−8.608 ^b^	3.056
MSN-TEDTH-PEI-FA-TR-Sn	−8.105 ^b^	2.834

^a^: In PBS and ^b^ in cell culture medium.

**Table 5:** IC_50_ data of the different synthesized nanomaterials against Hek 293T cell line.

Materials	IC_50_ (µM)	Metal [Sn] (µM)
MSN	>500	-
MSN-TEDTH-PEI-FA	>500	-
MSN-TEDTH-PEI-FA-MPP	>500	-
MSN-TEDTH-PEI-FA-TR	>500	-
MSN-TEDTH-PEI-FA-MPP-Sn	>500	>1.60
MSN-TEDTH-PEI-FA-TR-Sn	228.10	1.20

**Table 6:** IC_50_ data of the different synthesized nanomaterials against MCF-7 cell line.

Materials	IC_50_ (µM)	Metal [Sn] (µM)
MSN	>500	-
MSN-TEDTH-PEI-FA	397.90	-
MSN-TEDTH-PEI-FA-MPP	216.50	-
MSN-TEDTH-PEI-FA-TR	391.70	-
MSN-TEDTH-PEI-FA-MPP-Sn	246.48	0.78
MSN-TEDTH-PEI-FA-TR-Sn	23.97	0.13

## Data Availability

The data supporting the findings of this study are available from the corresponding author upon reasonable request.

## Supplementary Materials

The following supporting information can be downloaded at: https://www.mdpi.com/article/10.3390/nano15231791/s1, Figure S1. UV-vis spectra from the measurement of all the synthesized materials, showing the maximum absorption peaks of the compounds: (a) 200–450 nm; (b) 300–550 nm; Figure S2. FT-IR spectra of all the synthesized materials, showing all the bands belonging to the binding vibrations of the compounds of interest: (a) 4000–2500 cm^−1^; (b) 1800–450 cm^−1^; Figure S3. Nitrogen desorption-adsorption isotherms of materials (a) MSN; (b) MSN-TEDTH-PEI-FA-MPP-Sn; (c) MSN-TEDTH-PEI-FA-TR-Sn; Figure S4. TEM images of MSN; Figure S5. FEG-SEM elemental mapping showing the uniform distribution of O, Si, Sn, and N on the surface of MSN-TEDTH-PEI-FA-MPP-Sn; Table S1. Elemental composition of MSN-TEDTH-PEI-FA-MPP-Sn obtained by FEG-SEM; Figure S6. FEG-SEM images of MSN-TEDTH-PEI-FA-TR-Sn showing the spherical arrangement of the nanoparticles (4 figures up) elemental mapping showing the uniform distribution of O, Si, Sn, and N on the surface of MSN-TEDTH-PEI-FA-TR-Sn (down); Table S2. Elemental composition of MSN-TEDTH-PEI-FA-TR-Sn obtained by FEG-SEM; Table S3. Data obtained in the DLS mesurement of the final materials in biological medium.
