# Supplementary material for: Dual Synthetic Pathways for Organotin-Functionalized Mesoporous Silica Nanoparticles: Targeted Therapeutic Platforms with Folic Acid and PEI Formulation
Source: Nanomaterials (Basel). 2025 Nov 27;15(23):1791. doi: 10.3390/nano15231791 (PMC12692869; doi:10.3390/nano15231791)
Supplement: Supplementary file 1 [file nanomaterials-15-01791-s001.zip › nanomaterials-3926328-supplementary.pdf]

nanomaterials

MDPI

Dual Synthetic Pathways for Organotin-Functionalized Mesoporous Silica Nanoparticles: Targeted Therapeutic Platforms with Folic Acid and PEI Formulation

Victoria García-Almodóvar [1], Sanjiv Prashar [1,2] and Santiago Gómez-Ruiz [1,2,*]

[1] COMET-NANO Group, Departamento de Biología y Geología, Física y Química Inorgánica, E.S.C.E.T., Universidad Rey Juan Carlos, Calle Tulipán s/n, Móstoles, E-28933 Madrid, Spain
[2] Instituto de Investigación de Tecnologías para la Sostenibilidad, Universidad Rey Juan Carlos, Calle Tulipán s/n, Móstoles, E-28933 Madrid, Spain
* Correspondence: santiago.gomez@urjc.es

1. UV–visible spectroscopy studies (DR UV-vis)

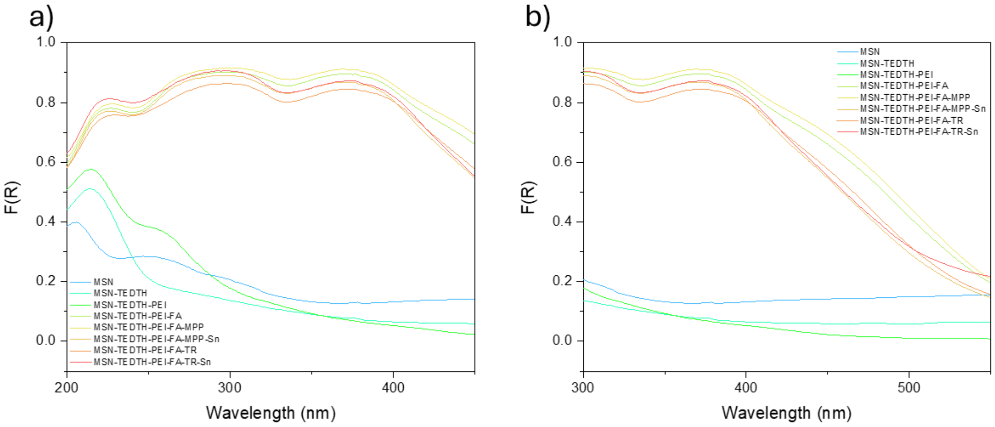

Figure S1. UV-vis spectra from the measurement of all the synthesized materials, showing the maximum absorption peaks of the compounds: (a) 200-450 nm; (b) 300-550 mn.

2. FT-IR studies

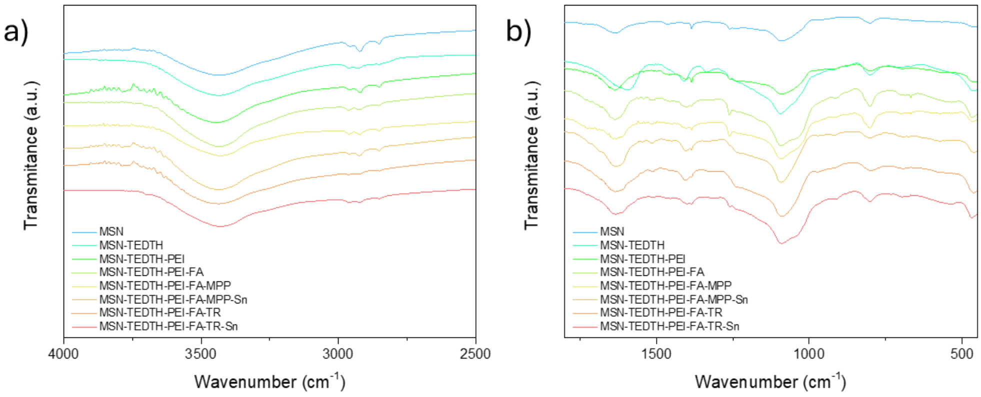

Figure S2. FT-IR spectra of all the synthesized materials, showing all the bands belonging to the binding vibrations of the compounds of interest: (a) 4000-2500 cm^{-1}; (b) 1800-450 cm^{-1}.

3. Nitrogen adsorption-desorption isotherms (BET)

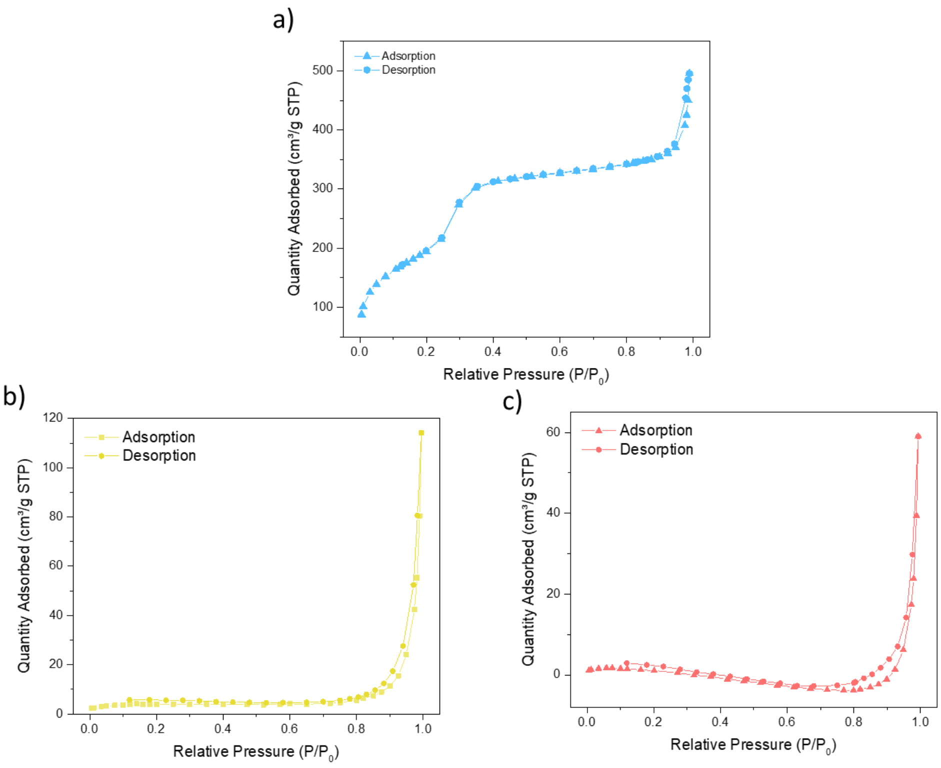

Figure S3. Nitrogen desorption-adsorption isotherms of materials (a) MSN; (b) MSN-TEDTH-PEI-FA-MPP-Sn; (c) MSN-TEDTH-PEI-FA-TR-Sn.

Figure S3 shows the BET isotherms of the three materials. As observed, the initial material exhibits the typical adsorption–desorption behaviour of mesoporous silica nanoparticles (MSNs), while the final materials display a markedly different profile. This variation can be attributed to the saturation of the pores and the successful functionalisation of the surface with a large molecule such as the PEI polymer, which precludes the canonical desorption of nitrogen.

4. Electronic microscopy studies (TEM)

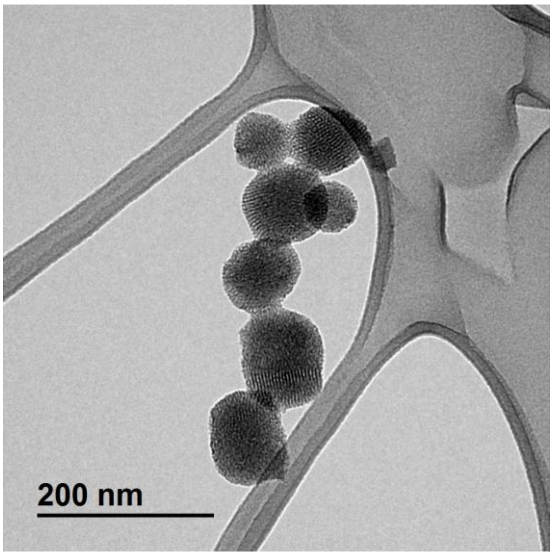

Figure S4. TEM images of MSN.

5. Field emission scanning electron microscopy (FEG-SEM)

Microstructural studies and semi-quantitative elemental analysis were conducted using an Apreo ChemiSem (Thermo Scientific) with energy dispersive X-ray spectrometer (EDS) at the Technological Support Center of the Rey Juan Carlos University (CAT). The images were captured by secondary electrons (LVD) and Backscatter Detector (ABS) with a beam energy of 5-10 kV. The magnifications were adjusted 28kX for all examined samples The operating parameters for elemental analysis were as follows: accelerating voltage, 10 kV; accumulation time, 60 s. The utilization of the low vacuum mode (50Pa) facilitated the examination of samples in their inherent state, devoid of any conductive coating, for the purpose of charge compensation.

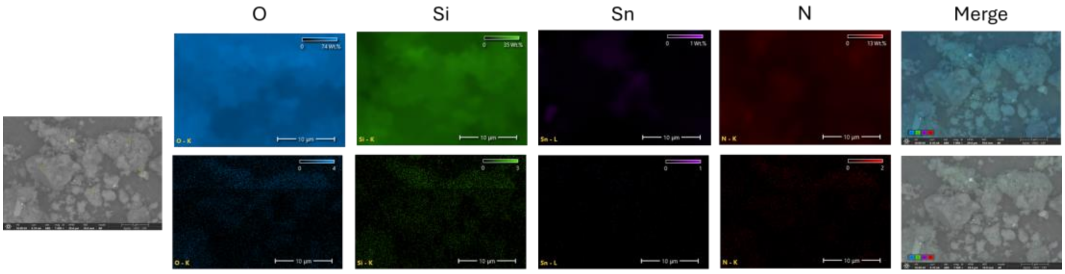

Figure S5. FEG-SEM elemental mapping showing the uniform distribution of O, Si, Sn, and N on the surface of MSN-TEDTH-PEI-FA-MPP-Sn.

Table S1. Elemental composition of MSN-TEDTH-PEI-FA-MPP-Sn obtained by FEG-SEM.

Element	At. (%)	Wt. (%)
N	12.1	9.4
O	70.4	62.2
Si	17.3	26.8
Sn	0.2	1.6

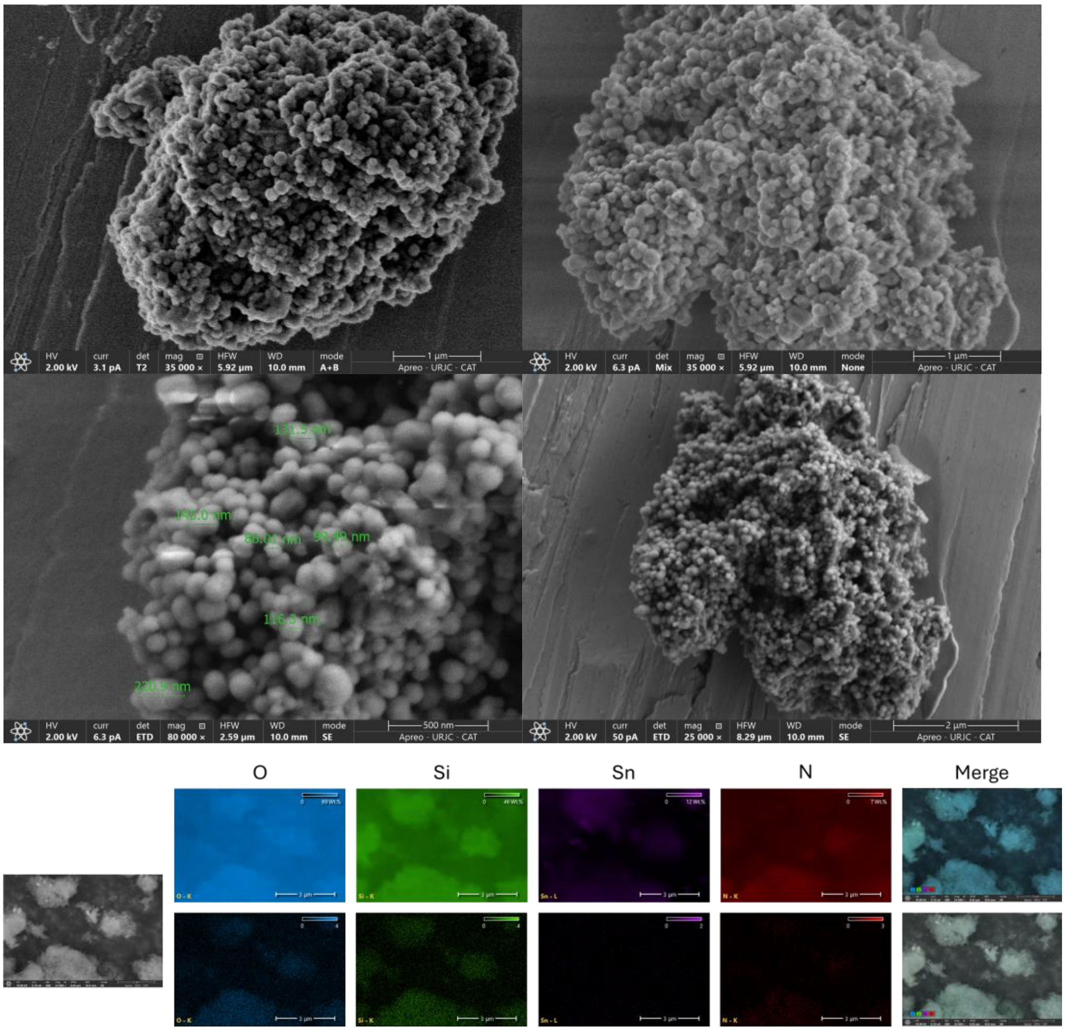

Figure S6. FEG-SEM images of MSN-TEDTH-PEI-FA-TR-Sn showing the spherical arrangement of the nanoparticles (4 figures up) elemental mapping showing the uniform distribution of O, Si, Sn, and N on the surface of MSN-TEDTH-PEI-FA-TR-Sn (down).

Table S2. Elemental composition of MSN-TEDTH-PEI-FA-TR-Sn obtained by FEG-SEM.

Element	At. (%)	Wt. (%)
N	11.8	9.1
O	69.6	61.2
Si	18.4	28.3
Sn	0.2	1.4

6. Z-Potential and Dynamic Light Scattering (DLS)

Table S3. Data obtained in the DLS mesurement of the final materials in biological medium.

Materials	Peak intensity (nm)
MSN-TEDTH-PEI-FA-MPP-Sn	138.44
MSN-TEDTH-PEI-FA-TR-Sn	206.79
